# Difficult Dentition and the Use of the Gum Lancet

**Published:** 1883-11

**Authors:** J. Morgan Howe


### Independent Practitioner.

VOL. V. NOVEMBER, 1883. NO. 11.

### Original Communications.

### DIFFICULT DENTITION AND THE USE OF THE GUM LANCET.

BY J. MORGAN HOWE, M. D., M. D. S.

Primary dentition has long been regarded as a cause of many infantile ailments. This view, based largely on clinical observation, has been that of some of the most eminent physiologists and practitioners, and has come to be so commonly accepted that it is quite probable that the origin of many disturbances has often been falsely attributed to teething, as it furnished a convenient scape-goat for indolence and ignorance. A just antagonism of the evil has perhaps been the pendulum rod on which some have swung over to the conclusion that it is all a mistake, to suppose that so simple a process is capable of producing any serious disorders of nutrition, or of functional activity ; and to fortify this position the fact that *dentition is a physiological process* is set forth, with apparent confidence that it should be sufficient to convince all disbelievers in this opinion. Although the use of this truth as basis for an argument has had force or plausibility enough to have caused its repetition by several writers, it hardly seems to be conclusive, and we propose to offer some facts and suggestions as reasons for believing that the deductions of so many, reached by clinical experience, that dentition is a cause of nervous irritation and of numerous reflex disturbances, is not a mistaken one; and in doing so, we quote from two writers who, from their position and special dental knowledge, may be the more likely—if their views are erroneous—to mislead.

In an address on Oral Surgery before the American Medical Association, 1882, Dr. D. H. Goodwillie in deprecating the fact that teething has often been given as the cause of death in mortuary certificates, says; "Alas! a normal physiologicalprocess the cause of death. If first dentition in the child is the cause of death, why not carry it through the process of second dentition, which lasts nearly to adult age? Perhaps it might be found that malaria, smallpox or some of the other diseases lie buried in the jaws." And Dr. W. C. Barrett in an editorial in the *Independent Practitioner*, March, 1883, says; "About the time when the molar teeth are in process of eruption, digestive ills of all sorts attack the child, and to the mere physiological process of cutting the teeth is attributed the flatulence, diarrhœa, convulsions and death," etc. * * "Digestion and assimilation being properly performed, there is nothing in the mere process of the eruption of the teeth which can cause any serious disturbance. Nature has provided for the absorption and disappearance of the tissues covering the growing teeth without any febrile symptoms, any diarrhœa or nervous convulsions;" and again he says; "The gums and investing tissues have not such an exquisitely nervous organization, are not so thoroughly supplied with nerve fibers as to produce any grave disturbances by the mere force of an advancing tooth. There is little or no sensitiveness in the raised gum. Pressure over the coming tooth is not annoying to the infant; on the contrary it often seems grateful. Whence then this idea of dangerous reflex nervous disturbances?"

It seems to have been Dr. Goodwillie's intention to ridicule the idea of dentition ever being a cause of death, basing the assumption of its absurdity on the fact that it is a physiological process, which he calls normal! Whether he meant to assert that it is always normal may be doubted. If that were true, it would be of value to the race as well as to his argument; if it is admitted that it is not always a *normal* physiological process, it would be interesting to know of how many other human physiological processes the claim would be made, that they could not be the cause of such irritation and disturbance as directly or indirectly to cause death. All the tissue changes and movements involved in the developmental processes of the human animal are so frequently attended with more or less manifestation of abnormality, that it is difficult to perceive why any one should assume that dentition is exempt from anomalies which so commonly cause other physiological processes to produce serious derangement of the system. Merely to mention gestation and parturition, is to call to mind morning sickness, eclampsia, post partem hemorrhage, and various other complications. There is abundant reason for regarding dentition as similar to these processes, in that it makes great demands upon the vitality of the subject, and that its expression is one of irritation during a considerable portion of its continuance. One of the earliest signs of the advancement of the partly calcified developing teeth, is increased salivation—drivelling; it is as rarely absent perhaps as the morning sickness of pregnancy, and seems without doubt to be the result of gland excitement, through the irritation of afferent nerve filaments in the tooth pulp. This phenomenon, generally accompanied with fretfulness and signs of pain, makes its appearance at the time (fifth to sixth month) * when the developing incisor teeth would be advancing toward or into the orifices of the alveolar crypts, the margins of which should be enlarging by absorption to admit of their passage; but in case of irregularity of the eruptive force, and lack of corresponding absorption of the obstructing tissues, pulp irritation would necessarily ensue. And we may well suppose, with any such anomalies, a greater or less degree of local hyperæmia. Similar reflex excitement of the salivary glands, by irritation of the filaments from the trigemini in the tooth pulp, is witnessed daily in the dental chair, the saliva being frequently thrown out in jets, upon instrumental or other irritation of the dentine, or dental pulp; and it is equally patent that dental irritation of whatever kind is generally attended with increase of saliva-

* Tome's Dental Surgery.

tion, the glands being excited much more readily by irritation of nerve filaments distributed to the deeper parts, than of those in the gum tissue, for the latter, as Dr. Barrett says, "have not such an exquisitely nervous organization."

In turning the attention to the development and eruption of the permanent teeth, we find no such disturbances of the system as have been generally attributed to teething in the infant subject; the conclusion is therefore not unnatural, that first dentition is not the baleful process it has been called. We think, however, it is sufficient to call attention to the fact that the period is one of peculiar susceptibility to derangement of the harmony of vital processes, from any cause. The liability of the alimentary tract to irritation from slight variations of food, from thermal and atmospheric impressions, is indicative of the liability of all the organs and tissues of thebody to be disturbed by predisposing or immediate influences, so that anomalous movements in the physiological mechanism are more likely to be produced, and to disturb the system when they occur. Dr. Barrett attributes to injudicious feeding almost all the ailments that have frequently been considered due to teething. It is one of the most common and important causes of infantile ills, but we think dentition is perhaps as common, and is equally as important a factor in producing those well known disturbances which so frequently endanger life at this time.

There are occasional instances in second dentition, during the efforts of nature to erupt the third molar, especially of the inferior jaw, of irritation which differs, if at all, only in degree from that which is commonly produced by the eruption of the deciduous teeth. This has occurred sometimes under our own observation, when, neither at the time nor after the appearance of the tooth, could any unusual position of the growing tooth or of surrounding parts be discovered, and no reason could be assigned, except a lack of absorption of the super-imposed mass of gum tissue, in response to the probably too fitful and irregular pressure of the tooth. Such irritation is, however, more frequently observed, when (1) the space between the posterior surface of the second molar and the compact bone of the ascending ramus is insufficient for the passage of the crown of the third molar, or (2) when the latter tooth is tipped anteriorly, so that its progress is arrested and the eruptive force is expended on the posterior surface of the second molar. Such complications are not very uncommon, and from them frequently arise reflex neuralgias of the severest nature, often with fever. Without detailed subjective symptoms these cases would afford fruitful ground for difference of opinion, for in many there are no local indications of inflammation, either of the soft tissue around the developing tooth or of the second molar, and no soreness of the gum or of the growing tooth to pressure ; and in other cases, principally those referred to, even when a considerable degree of inflammation in the soft tissues is present, the local conditions seldom if ever appear adequate to an explanation of the severe reflex neuralgia from which the patient suffers. We would be very likely to look elsewhere for a cause of such violent distress as frequently occurs, rather than to a developing tooth, if the patient's own apprehension of the cause were taken from us. During first dentition we have no subjective symptoms to aid us in the formation of diagnoses, and these cases of difficult eruption of third molars may well be considered fair illustrations of the irritation possible from all impeded dental growth. Neuralgia is generally the form of greatest suffering in these cases of anomalous wisdom teeth, the pains often extending to the head and neck, and sometimes to the arms, although it is not confined to this, for the nutrition of other organs and tissues may be disturbed. Among those who have observed the phenomena produced by these anomalous teeth of second dentition, there are probably few who have regarded the irritation of the obstructing tissue as the cause of the most serious symptoms ; the majority would be likely to attribute the disturbances to their true cause, viz: *pulp irritation.* There seems to be abundant reason for regarding the pulp as the point of irritation of afferent nerves when reflex actions are produced, whether it occur through obstruction to the growth of a wisdom tooth, or the deciduous teeth. In the latter it is quite true that there is no anomaly of arrangement worth considering, and "nature has provided for the absorption and disappearance of the tissues covering the growing tooth," but we see no more reason for claiming that this is accomplished in perfect ratio with all the other movements involved, than that all other physiological processes are accomplished in a perfectly normal manner. When the possibilities, in the direction of nervous disturbance, from an irritated pulp are considered, argument against dentitional irritation, because the gum is not an exquisitely sensitive tissue, seems hardly convincing. When the susceptibility to derangement and to nervous irritation of the infant be considered, the pulp irritation of anomalous wisdom teeth may, we think, give us some light on the sufferings sometimes endured by the little ones. The irritation so frequently produced by first dentition begins, as we have endeavored to show, at an early period, and may cause serious reflex disturbances at any time thereafter. The emesis, diarrhœa, and convulsions of this period, are of course liable to be produced by other causes, but the clinical experiences of numerous competent practitioners has been amply sufficient to confirm the old opinion, that these phenomena are often due to dental irritation, so that the conclusion will hardly be disturbed by argument based on the fact that dentition is a physiological process, nor by negative testimony from those who have not observed such ailments under circumstances which have convinced them.

It must be admitted that positive and satisfactory diagnoses are not always easily formed, but the difficulty is probably not greater in these cases than in most others where subjective symptoms are wanting. There are many instances which appear to indicate dental irritation so clearly as to hardly leave room for doubt. Such are those in which nursing infants occasionally give sudden manifestations of distress, with gastric, intestinal or cerebral disturbance, without other assignable cause; there is increased salivation, and the child not unfrequently indicates in various ways that the seat of pain is in the mouth. All this ismuch more likely to occur in the case of infants who do not nurse, but it cannot fairly be attributed to improper feeding when the child has been thriving on the same food up to the period of such disturbance. Such cases have occurred, and are appearing more or less constantly, with numerous variations of circumstance. The literature of this subject is very meager, but Dr. Samuel Sexton * has called attention to the fact that some aural diseases have their origin in dental irritation by reflex action, and has graphically shown how this takes place through the Otic ganglion. He has clinically recognized the fact that the processes of dentition, especially of the deciduous teeth and the wisdom teeth, often cause this irritation, as well as dental caries, peridentitis, and alveolar abscess.

It is not our present purpose to endeavor to indicate the avenues by which the numerous reflex actions from dental irritation are accomplished ; they are probably often of a very complicated nature, but I will merely call attention to the direct connection of the trigemini with four ganglia and one plexus of the sympathetic system, whose controlling influence over vascular supply and gland action is so well recognized, and to the comparatively simple manner by which cerebral circulation may be influenced through the vaso motor nerves of the internal carotid artery and its encranial branches, through the connection of the fifth pair of nerves with the Otic ganglion and the carotid plexus.

The treatment of cases giving evidence of difficult primary dentition should of course be governed by a careful consideration of that process itself, as well as the character and severity of the symptoms manifested in each individual case.

Whatever means give reasonable promise of adding tone to the system, may cause the disappearance of reflex symptoms, whether produced by dental irritation or other causes. Hygienic measures, especially if these have been neglected, are always to be considered. High temperatures have a general debilitating influence, and on this account reflex phenomena are more easily provoked under these conditions, a change of location giving lower temperature and other favorable circumstances often proving beneficial.† Change of diet may be indicated, and therapeutic measures judiciously directed to meet the symptoms of the case will often be all that is needed to cause the latter to subside, and enable the processes of development to progress more harmoniously.

* American Journal Medical Sciences, January, 1880; also circular of information of the Bureau of Education, No. 5, Washington Government Printing Office, 1881.

†(Brown-Sequard, Cayrade.) Kuss Lectures on Physiology, p. 54.

There are instances, however, in which all such means are insufficient, affording at best but partial and transitory relief; the child gives frequent evidences of oral suffering, or of nervous irritation by reflex disturbances; in some such cases the gum lancet affords the means of rendering the most prompt and efficient relief. Its use is clearly counter-indicated in such early stages of teething as when the advancing teeth are probably obstructed by alveolar tissue, but when the enlarged gum indicates both to sight and feeling the presence of the tooth beneath it, when the former tissue has a tense appearance (whether it is sore, swollen and red, or not), with the disturbances before referred to, which more general treatment has failed to relieve, a free incision through the gum to the tooth, with a sharp lancet, will in most instances be promptly followed by very marked amelioration of all symptoms of irritation. We have repeatedly performed this trifling operation with such salutary results, in very few instances without them. The ineffectual use of the lancet may prove a mistaken diagnosis, or that the irritation proceeded from teeth less advanced in development than those released by the incision; it does not prove that dentition is incapable of producing irritation which may, through reflex action, endanger life. The instances, however, in which gum lancing is not followed by relief, *when it has been indicated* by the conditions and symptoms, are so rare that it may be regarded as one of the most certain and effectual of minor operations, and so far as we know is contra-indicated only by a hemorrhagic diathesis.

The objection, urged sometimes, that the gum will be made much harder (if it should heal) by the formation of a cicatrix, and the temporary relief hoped for be followed by an aggravation of the difficulty of absorption of the gum, is invalid, from the fact that a cicatrix is not found after gum lancing, and if there should be a formation of cicatricial tissue it would absorb more readily than the primary tissue.

The valid objection to the lancing of the gums of teething children is the "almost indiscriminate" practice of it, which Dr. Barrett believes is "falling into desuetude." There are probably few who will not be glad with him, and hope that all other practices that approach the indiscriminate may find the same limbo; but judicious gum lancing, practiced with discrimination and judgment, not for the purpose of depleting a congested gum but to release an imprisoned or obstructed tooth, is both reasonable and commendable, and its value should not be overlooked.

Cases of obstructed eruption of wisdom teeth often demand local treatment.

Those whose position in the angle between the body and ramus of the jaw causes them to be covered with a mass of soft tissue, which is not absorbed sufficiently to prevent it becoming inflamed by the pressure of the growing tooth and of antagonizing teeth in the superior jaw, may be made less troublesome by proper lancing, but permanent relief is only obtained, in many instances, by the removal of the tooth for which nature has failed to provide a place.

Extraction must also frequently be resorted to when neuralgic or other disturbances arise through obstruction of the wisdom tooth by the second molar, or by the latter together with the maxillary ramus, or when the former is tipped forward so that its progress is arrested by the second molar. When the third molar occupies so nearly a horizontal position that the crown is in contact with the neck of the second molar, the extraction of the former is often an impossibility, without resorting to an entirely inexpedient operation, and in such case the removal of the second molar, the obstructing tissue, must be chosen as the least evil.

But wisdom teeth placed anomalously in the maxillæ not unfrequently attain to complete development before meeting in their advance such resistance as to cause pulp irritation with local and reflex disturbance, and as interference is not demanded, it would unquestionably seem to be a blunder.

---

### ANÆSTHESIA.

The experiments of M. Paul Bert show that the method of administering chloroform is best and least dangerous by which the patient is quickly anæsthetized by a large quantity, and then kept under the anæsthetic by a much smaller amount.—*L' Union Med.*

